# Feasibility, Acceptability, and Design of a Mobile Ecological Momentary Assessment for High-Risk Men Who Have Sex With Men in Hanoi, Vietnam: Qualitative Study

**DOI:** 10.2196/30360

**Published:** 2022-01-27

**Authors:** Kathy Trang, Lam X Le, Carolyn A Brown, Margaret Q To, Patrick S Sullivan, Tanja Jovanovic, Carol M Worthman, Le Minh Giang

**Affiliations:** 1 Global TIES for Children New York University New York City, NY United States; 2 Vietnam National University Hanoi Vietnam; 3 Amgen Santa Monica, CA United States; 4 Department of Psychiatry and Behavioral Sciences School of Medicine Emory University Atlanta, GA United States; 5 Department of Epidemiology Rollins School of Public Health Emory University Atlanta, GA United States; 6 Department of Psychiatry and Behavioral Neuroscience School of Medicine Wayne State University Detroit, MI United States; 7 Department of Anthropology Emory University Atlanta, GA United States; 8 Department of Epidemiology Hanoi Medical University Hanoi Vietnam

**Keywords:** men who have sex with men, HIV, mental disorder, ecological momentary assessment, mobile phone, mHealth, sexual minorities, pilot projects

## Abstract

**Background:**

Men who have sex with men (MSM) are at a disproportionate risk for HIV infection and common mental disorders worldwide. In the context of HIV, common mental disorders are important and are frequent drivers of suboptimal prevention and treatment outcomes. Mobile ecological momentary assessments (EMAs), or the repeated sampling of people’s behaviors and psychological states in their daily lives using mobile phones, can clarify the triggers and HIV-related sequelae of depressive-anxious symptoms and contribute toward the design of ecological momentary interventions (EMIs) that cater to the contextually varying needs of individuals to optimize prevention and treatment outcomes.

**Objective:**

This study aims to characterize the feasibility and acceptability of mobile EMA among high-risk MSM in Hanoi, Vietnam. It aims to evaluate the perceived relevance, usability, and concerns of this group with regard to the content and delivery of mobile EMA and the potential of leveraging such platforms in the future to deliver EMIs.

**Methods:**

Between January and April 2018, a total of 46 participants were recruited. The participants completed 6 to 8 mobile EMA surveys daily for 7 days. Surveys occurred once upon waking, 4 to 6 times throughout the day, and once before sleeping. All surveys queried participants’ perceived safety, social interactions, psychological state, and mental health symptoms. The morning survey further queried on sleep and medication use within the past 24 hours, whereas the night survey queried on sexual activity and substance use and allowed participants to share an audio recording of a stressful experience they had that day. At the end of the week, participants were interviewed about their experiences with using the app.

**Results:**

Participants completed an average of 21.7 (SD 12.7) prompts over the 7-day period. Excluding nonresponders, the average compliance rate was 61.8% (SD 26.6%). A thematic analysis of qualitative interviews suggested an overall positive reception of the app and 5 recurring themes, which were centered on the relevance of psychological and behavioral items to daily experiences (eg, mental health symptoms and audio recording), benefits of using the app (eg, increased self-understanding), worries and concerns (eg, privacy), usability (eg, confusion about the interface), and recommendations for future design (eg, integrating more open-ended questions).

**Conclusions:**

Mobile EMA is feasible and acceptable among young MSM in Vietnam; however, more research is needed to adapt EMA protocols to this context and enhance compliance. Most participants eagerly provided information about their mental health status and daily activities. As several participants looked toward the app for further mental health and psychosocial support, EMIs have the potential to reduce HIV and mental health comorbidity among MSM.

## Introduction

### Background

Approximately one-third of people living with HIV worldwide meet the criteria for a common mental disorder (CMD), such as depression and anxiety [1-4]. Among men who have sex with men (MSM) specifically, the prevalence of CMDs may be magnified, with an estimated 35% reporting a history of suicidal ideation worldwide [5], 43% living with HIV reporting depression [6], and at least one-third in the United States meeting criteria for past-year alcohol use disorder [7]. The pronounced stigma, vulnerability to intimate partner violence, discrimination, and criminalization of same-sex sexual activities that this group confronts worldwide may have multiplicative effects on the development and maintenance of CMDs [8-10]. In the context of HIV, CMDs are the important and frequent drivers of suboptimal prevention and treatment outcomes. Depression has been found to be associated with a reduced likelihood of initiating antiretroviral therapy (ART) [11], lower ART adherence [12-14], increased mortality risk [15], and accelerated HIV progression [16]. Although anxiety disorders, including posttraumatic stress disorder (PTSD), have been less frequently investigated in the context of HIV, growing evidence suggests that they can reduce the likelihood of achieving at least 80% adherence [17,18]. Anxiety may also increase sexual transmission risk behaviors, suicidal ideation, and cognitive impairments [2]. Among those HIV–, CMDs are linked to hazardous drinking [19], inconsistent condom use [20-22], and lower pre-exposure prophylaxis (PrEP) adherence [23], all of which increase the risk of HIV transmission.

Smartphones and other information and communication technologies have become increasingly attractive options for optimizing prevention and treatment strategies. Given their accessibility, affordability, and availability, these digital technologies promise to widen access to health care generally and specifically among vulnerable groups by lowering costs and the traditional barriers (eg, stigma and access) hindering participation. Mobile health (mHealth), or the delivery of interventions and other health services via digital technologies, can take the form of SMS text messages, multimedia messages, mobile apps, and social media campaigns, among other options. mHealth interventions have shown promising results in improving medication adherence [24,25], health care engagement [24,26,27], and other health-promoting behaviors such as engagement in physical activity [28-30] in diverse populations. Furthermore, mHealth may be an acceptable and feasible approach for delivering care, including mental health care, to MSM both in high-income countries and low- and middle-income countries (LMICs), including Malaysia, China, India, Thailand, and Vietnam [31-40]. This is because MSM are often early adopters of technology and may already use such platforms to access health information and search for sexual partners [34,41]. A recent survey of young MSM between the ages of 18 and 24 years found that approximately 70% were willing to participate in a web-based or mobile HIV prevention program, whereas only 1% would attend in-person programs exclusively [33]. As the access to and use of digital technologies do not appear to differ by mental health burden or HIV risk [42,43], mHealth may be a particularly attractive option for engaging underrepresented groups in settings where services are scarce.

Given the heterogeneity of risk among MSM [44-46], mHealth may further enable the tailoring of mental health and psychosocial interventions to the members’ individually varying needs. However, one of the key limitations of the literature on mental health and HIV acquisition risks and treatment is its reliance on cross-sectional data, whereupon participants are tasked to recall symptoms or behaviors unfolding weeks or months prior. Recall can bias individuals to particular events, for instance, by increasing the salience of negative memories when depressed [47], modifying the memory of past judgments when providing new information [48], and skewing estimates of symptom severity based on its peak or most recent occurrences [49]. Similarly, although these data allow for estimations of an event frequency (eg, an experience of heightened depressive symptoms or a sexual encounter), they provide little information about the contextual factors leading up to, during, or after the event, that is, on the triggers and HIV-related sequelae of depressive-anxious symptom experience.

Mobile ecological momentary assessments (mEMAs), or the repeated sampling of individuals in real time during the flow of their everyday lives using mobile phones, can reduce the impact of these biases and provide the high-resolution data necessary to model these relationships longitudinally *within* an individual. Comparisons of ecological momentary assessment (EMA) to retrospective approaches using calendars or audio and computer–assisted self-interviews have suggested that the retrospective approaches may contribute to the underestimation of sexual and substance use behaviors by as much as 50% and lead to distortions in specific details, including partner characteristics [50,51]. Comparing EMA reports of patients’ experience of side effects associated with antidepressant use with their reports to their general practitioner, 1 study found that although 43% of the patients reported experiencing dizziness in the moment, <20% of them reported this to their general practitioner [52]. By clearly delineating the relationships between mental health and HIV risk-taking behaviors, mEMA approaches may thus contribute to a scientific understanding of what drives differentials in temporary and long-term treatment compliance, outcome, and cessation. This can inform the design of ecological momentary interventions (EMIs) that cater to the dynamically changing risks of individuals across time and context.

### Objective

Although many mHealth studies have been implemented in LMICs [53], to our knowledge, only 1 mEMA study has been conducted in an LMIC. Specifically, Soong et al [54] evaluated the feasibility and acceptability of monitoring tobacco use in urban India and found lower compliance (46%) than is typical in substance use research conducted in high-income countries (65%-92%). This suggests the need to evaluate how EMA protocols can be adapted to low-resource settings and particularly vulnerable populations to maximize their feasibility, acceptability, and usability for future intervention design. Although there is a lack of consensus on how such protocols should be culturally adapted, qualitative methods can be particularly beneficial in understanding the target population’s needs and contexts of use. Integration of the target population’s input into the design process can increase the perceived ownership of the technology and optimize the relevancy, uptake, and eventual utility of the platform. Thus, this study sought to determine the feasibility and acceptability of an mEMA app focused on the behavioral and psychosocial linkages between mental health and HIV-related behaviors among high-risk MSM in Hanoi, Vietnam. In Vietnam, MSM continue to experience significant HIV and mental health burdens amidst decline among other key populations [55-58]. The findings from this study can optimize the design of future mEMA protocols and lay the foundation for EMIs tailored to this population.

## Methods

### Study Design

This study recruited participants from an existing pool of MSM (n=198) who had previously participated in a study examining the association between HIV and PTSD in Hanoi, Vietnam [59]. All participants in the original sample were recruited from sexual health clinics and community-based organizations in Hanoi, were between the ages of 18 and 29, had reported having engaged in anal intercourse with a same-sex partner within the past 6 months, and had a smartphone.

From the existing pool of participants who had agreed to be recontacted (190/198, 96%), a subsample was derived based on the HIV serostatus and probable PTSD diagnosis of individuals, which was indicated by their scores on the Modified Posttraumatic Stress Scale [60]. The criteria for PTSD are dependent on the presence of a criterion A trauma (death, the possibility of death, or serious bodily harm, or actual or threatened sexual violence) alongside at least one intrusive symptom, three avoidance or numbing symptoms, and two hyperarousal symptoms that have been present for at least one month, as indicated on the Modified Posttraumatic Stress Scale. A total of 4 PTSD-HIV strata (PTSD+/HIV+, PTSD+/HIV–, PTSD–/HIV+, and PTSD–/HIV–) were created, and a list of individuals belonging to each group was randomly generated. Individuals were recontacted in that order.

**Figure 1:**
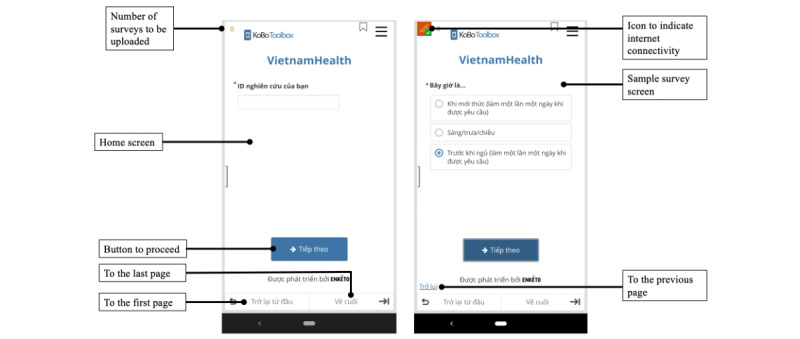
Screenshots of the mobile ecological logical momentary app that was powered by Enketo and piloted among young men who have sex with men (N=46) in Hanoi, Vietnam.

Upon enrollment, participants completed assessments of their mental health and sexual and drug use history. The study staff then instructed the participants on how to install a secure web app enabling real-time data collection, KoBoToolbox [61], on their smartphones. Briefly, the participants were first added to the KoBo system and then sent a link to download the web app onto their phone, where it could then be accessed. The web app was powered by Enketo and specifically designed for low-resource settings. Other countries where KoBo has previously been implemented include Vietnam, Indonesia, Ghana, and Malawi [62,63]. This platform was selected as it was free and open source, adaptative in a range of challenging contexts with varying Wi-Fi availability, and had accessible features that could be further expanded and customized depending on user feedback during the exit interview. Data collected through KoBo were stored on a secure server hosted by Amazon Web Services, which provided network and infrastructure security and monitored host and end point security. Only the study staff had access to the data. Screenshots of the app are provided in Figure 1.

After KoBo was installed, the participants were asked to complete a sample survey. The study staff were available to answer any questions or concerns. During the next 7 days, the participants were prompted to complete 6 to 8 surveys daily: 1 survey every morning upon waking (morning survey), 4 to 6 surveys at random time points throughout the day (midday survey), and 1 survey before sleeping (night survey). Most prompts occurred during midday. This decision was motivated by our scientific interest in understanding the dynamic interactions between PTSD and HIV-related risk-taking behaviors. Specifically, one of the features of PTSD is emotional lability or intense, unpredictable, and frequent shifts in emotional experience [64]. Emotional lability can hinder social relationships and functioning and contribute toward risk-taking behaviors. Repeated sampling of emotions, symptom experiences, and social experiences throughout the day would enable us to characterize emotional lability and the trajectory of symptoms and affective experiences leading up to a risk-taking episode. Through this study, we thus aim to test the acceptability and feasibility of such a granular measure of daily experience among a high-risk population.

Prompts were structured around participant-provided schedules and delivered randomly in 1- to 3-hour windows; the windows were approximately 30 minutes apart (eg, 7:30 AM to 9 AM and 9:30 AM to noon). With each prompt, participants received an SMS text message, reminding them to complete their survey on KoBo within the next 30 minutes. If the participants were unable to respond within that time frame, they were instructed to wait until the next prompt. Each morning and night survey could be completed in approximately 3 to 7 minutes, whereas midday surveys were completed in approximately 3 to 5 minutes. Prompts were uploaded automatically upon completion if internet access was available; otherwise, they were stored locally on the participants’ smartphones.

At the exit session, participants were interviewed about their experiences using the app, including their motivations for participating, perceived benefits or drawbacks of using the app, any worries or concerns they experienced, and their recommendations for future research. Interviews were semistructured and conducted in Vietnamese by trained research staff. Questions were open ended and intended to elicit feedback on the content, appearance, and functionality of the app. Participants were compensated 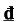
150,000 (US $6.45) for completing the exit interview, which averaged 20.4 (SD 17.1) minutes. For each EMA survey they completed, the participants were additionally compensated 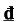
7000 (US $0.30). The maximum amount individuals could be compensated for participation in the EMA component of the study was 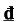
350,000 (US $15.05).

### Ethical Considerations

The study was approved by the institutional review boards of Emory University (approval number: IRB00097736) and Hanoi Medical University (approval number: IRB00088463). All participants were provided detailed information about the study procedures and expectations, risks, and benefits before their providing of written consent. The participants were told that they could choose to discontinue the mobile surveys at any point.

### Measures

In all surveys (morning, midday, and night), participants answered a standard set of questions. At each survey, the participants reported their current location, including perceived safety, and number of social interactions since the last prompt. For their most recent interaction, participants were asked to identify their relationship to that individual (eg, friend or sibling) and rate the ease of the interaction, the quality of the interaction, and their perception of what the other person had thought of them on a 7-point Likert scale. In addition, participants were asked to rate the degree to which they felt safe on a 6-point Likert scale and report whether they had experienced a range of PTSD and somatic symptoms (*yes* or *no*) within the past hour. Emotional states were assessed by asking the participants to rate the intensity of their emotions using the Positive and Negative Affect Schedule [65]. In only the morning survey, the participants were asked about their sleep (eg, times and quality) and ART or PrEP use within the past 24 hours.

For the night survey, sexual and drug use behaviors and desires within the past 24 hours were additionally assessed. For each reported sexual activity, the participants answered questions about the sexual act (eg, anal and oral), the sex of the partner, the nature of their relationship (eg, stable and casual), condom use, and drug use during sex. Finally, in the night survey, the participants were asked to recollect, in an audio recording, an event within the past 24 hours that made them tired, anxious, or stressed about the future. They were instructed to freely associate, in detail, the potential causes, consequences, or scenarios associated with this event, including their feelings about what has occurred or could have occurred. Table 1 shows how these different modules were distributed across the morning, midday, and night surveys.

**Table 1:** Modules in the morning, midday, and night surveys administered to young men who have sex with men (N=46) in Hanoi, Vietnam, between January and April 2019.

Variables	Morning prompt	Midday prompt	End-of-day prompt
Time	✓	✓	✓
Location	✓	✓	✓
Whether someone else is present and their identity	✓	✓	✓
In their most recent interaction, the quality of interaction	✓	✓	✓
In their most recent interaction, the ease of interaction	✓	✓	✓
In their most recent interaction, perception of what the person thought of them	✓	✓	✓
Perception of the level of safety at the time of the survey	✓	✓	
Time they went to sleep and woke up	✓		
Self-rated quality of sleep and current tiredness level	✓		
Experience of different emotions (eg, anger) and somatic symptoms (eg, dizziness) within the past hour	✓	✓	
Experience of difficulty in concentrating or regulating emotions or behaviors within the past 24 hours	✓	✓	
Use of PrEP^a^ or ART^b^ within the past 24 hours	✓	✓	
Level of stress at the moment and cause		✓	
Experience of different PTSD^c^ symptoms since the last prompt		✓	
Desire for different substances since the last prompt		✓	
Use of any substances since the last prompt		✓	
Experience of different emotions (eg, anger) and somatic symptoms (eg, dizziness) at the moment			✓
Experience of different PTSD symptoms within the past hour			✓
Desire for different substances at the moment			✓
Sexual activity within the past 24 hours			✓
Substance use within the past 24 hours			✓
Exposure to drugs or other substances			✓
Stressful experience within the last 24 hours (audio recorded)			✓

^a^PrEP: pre-exposure prophylaxis.

^b^ART: antiretroviral therapy.

^c^PTSD: posttraumatic stress disorder.

Measures of PTSD and somatic symptoms, as well as Positive and Negative Affect Schedule, had previously been culturally adapted using the 5-step translation process by van Ommeren et al [66]: (1) translation, (2) review by mental health professionals, (3) focus group discussions, (4) back translation, and (5) pilot of the measures among MSM in Hanoi, Vietnam. This 5-step approach has been used in several LMICs to maximize conceptual, semantic, technical, and content equivalence of measures in a new population or context [67]. Other items on the surveys were forward (English to Vietnamese) and backward (Vietnamese to English) translated by 3 bilingual translators.

### Qualitative Data Analysis

Interviews from the exit session were transcribed verbatim and anonymized by 2 research assistants and then imported into MaxQDA (VERBI), a qualitative data analysis software. Using content analysis [68], 2 authors (KT and LXL), who were bilingual, then independently reviewed the transcripts and generated a preliminary codebook before meeting to refine primary and secondary code definitions. The codebook contained both inductive and deductive codes. Coding was guided by the Unified Theory of Acceptance and Use of Technology model, which posits that adoption of technology is dependent on (1) performance expectancy or perceived benefit of using the technology; (2) perceived effort or ease of use; (3) social influences, including norms around the social acceptability of the technology; and (4) facilitating conditions or availability of technological, cultural, or organizational resources supporting technology use [69]. Of particular interest to coding was feedback on existing study design and questions, including the perceived relevance of each in the daily lives of the participants, and recommendations for future research design. Once the codebook was established, 2 analysts coded each transcript. The two met regularly to refine the codebook, discuss emergent themes, and resolve any disagreements about coding. After all transcripts were coded, the authors (KT and LXL) reviewed the excerpts to identify salient themes and select quotations that illustrated the major themes and subthemes.

## Results

### Participant Characteristics

Of the 198 participants in the original sample, 50 (25.3% of the original sample; 50/73, 69% of those invited) returned to complete this study between January and April 2019. The four groups differed in their participation rate: 95% (19/20) of those in the PTSD–/HIV– group who were invited to participate did, 67% (8/12) among the PTSD+/HIV– group, 54% (6/11) among the PTSD–/HIV+ group, and 57% (17/30) among the PTSD+/HIV+ group. Among those who did not participate, 40% (9/23) had moved and were no longer in Hanoi, 30% (7/23) could not be reached, and the remaining 30% (7/23) refused. Among those who agreed to participate, later, 4 participants (n=3, 75% PTSD+/HIV+ and n=1, 25% PTSD+/HIV–) withdrew from the study after the initial interview because of personal reasons (eg, too busy with work), leaving a final analytical sample size of 46.

The sociodemographic characteristics of the participants are summarized in Table 2. On average, the participants were aged 23.5 (SD 2.5) years. Approximately two-thirds had some university education. Most of the participants (35/46, 76%) earned >
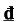
35 million or more (>US $1509) annually, which was considered the minimum needed to survive in Hanoi, where the average annual salary was approximately 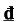
53 million (US $2337) to 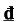
7,600,000 (US $3351) [70], which approximately 59% (27/46) of our participants either met or exceeded. In the sample, 41% (19/46) had neither PTSD nor HIV, 13% (6/46) were HIV+ and without PTSD, 15% (7/46) were HIV– and with PTSD, and 30% (14/46) had both HIV and PTSD. Among those with PTSD, the mean score on the PTSD Symptom Scale was 30.3 (SD 11.0), whereas, among those without PTSD, the mean was 12.4 (SD 7.9).

**Table 2:** Sociodemographic characteristics of men who have sex with men who participated in the mobile ecological momentary assessment study (N=46).

Variables		Values
**Sex, n (%)**		
	Male	46 (100)
**Age (years), n (%)**		
	18-22	14 (30)
	22-26	27 (59)
	26-29	5 (11)
**Migrant, n (%)**		
	Yes	26 (57)
	No	20 (44)
**Education, n (%)**		
	Some high school	5 (3)
	High school graduate	49 (25)
	Some university	74 (38)
	Graduated university	69 (35)
**Annual salary ( 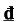 ; US $), n (%)**		
	0-35 million (0-1542)	11 (24)
	35-55 million (1542-2424)	8 (17)
	55-75 million (2424-3305)	14 (30)
	75-100 million (3305-4407)	8 (17)
	≥100 million (4407)	5 (11)
**PSS^a^ score, mean (SD)**		
	Total	20.6 (12.9)
	PTSD^b^–/HIV–	12.6 (8.6)
	PTSD+/HIV–	34.0 (13.0)
	PTSD–/HIV+	11.7 (5.8)
	PTSD+/HIV+	28.4 (9.8)

^a^PSS: Posttraumatic Stress Scale.

^b^PTSD: posttraumatic stress disorder.

### Compliance

Participants completed an average of 6.9 (SD 1.1) days of EMA activity reporting. During this time, they each received approximately 38.3 (SD 7.8) prompts and completed an average of 21.7 (SD 12.7) prompts. The average response rate was 56.5% (SD 30.9%) but ranged from 0% to 97.4%. Excluding individuals who did not complete any surveys (4/46, 9%), the average compliance rate was 61.8% (SD 26.6%). Among these individuals, the response rates were 64.5% (SD 33.1%) on the first day, 67.8% (SD 31%) on the second day, 66.9% (SD 31.6%) on the third day, 71.6% (SD 32.7%) on the fourth day, 64.7% (SD 35.6%) on the fifth day, 61.8% (SD 33.8%) on the sixth day, and 37% (SD 41.9%) on the seventh day. Demographics, group membership (based on HIV and probable PTSD diagnosis), and the number of prompts the participants received were not significantly associated with the response rate. Table 3 depicts representative quotes of each qualitative theme identified across groups of varying levels of compliance (75%-100%, 50%-75%, and <50%).

**Table 3:** Representative quotes from men who have sex with men participants with differing levels of ecological momentary assessment compliance.

Themes	High compliance (response rate >75%)	Medium compliance (response rate ≥50% and ≤75%)	Low compliance (response rate <50%)
Relevance	“[The relevant items] were about emotions, about mental health. [They] allowed me to understand what I have gone through, the mental state I had, and how they were affected by the stress I was experiencing in my life. [They] allowed me to better understand myself, my emotions as they really were.” [22 years, PTSD^a^+/HIV+]	“I think the questions that were most relevant had to do had to do with whether I was around someone at that time and whether I was satisfied with that interaction. Generally, it allowed me reassess the person.” [20 years, PTSD–/HIV–]	“[The most relevant question] was whether I had used my medicine, the question before I went to sleep. It’s relevant because, one, every day I must take medicine. Two, it’s also quite beneficial because if there are days where I forget, forget to take my medicine—although that’s very unlikely to happen—but I think if I were to use the app over a longer period, it could also help remind me on the days I would have otherwise forgotten.” [24 years, PTSD+/HIV+]
Benefits	“There are benefits. For example, I can track when I go to sleep, when I rest, when I wake, and what I do every day. I can assess whether it’s appropriate, appropriate for my work situation.” [23 years, PTSD–/HIV–]	“When you are answering the questions, it’s as though you are interrogating yourself about your own emotions at a particular moment...In general, I felt that I became more honest, more honest with myself. I asked myself what emotions I had, and I answered [that question]. I felt it allowed me to listen to myself more.” [21 years, PTSD–/HIV–] “I don’t know if there were any. I just felt the same.” [20 years, PTSD–/HIV–]	“Sometimes when I am doing the surveys, I realized that there were certain experiences I had that I hadn’t noticed before. For example, in the past hour, I might have lost my calm or felt anxious...Whenever I receive a prompt, I’d do the survey and I’d remember and think, yes, I did encounter that.” [23 years, PTSD+/HIV–] “To be honest, I don’t think there are any benefits because [the surveys] just remind me [of what happened]. It’s like writing a diary at the end of the day; there’s nothing new.” [24 years, PTSD+/HIV+]
Worries and concerns	“No, because really it’s just like a test or survey. There’s no reason for worry or hesitance. It’s like, like, you’re monitoring your own health.” [25 years, PTSD+/HIV+] “Sometimes the number of assessments were bothersome, but aside from when I am working, it wasn’t really a problem. Meaning, when the surveys were during school hours, when I was commuting, or when I had some tasks where I need to concrete, they were a little bothersome. But I felt that since I agreed to participate in this research, [fulfilling the surveys] was my responsibility, so I wanted to do everything to fulfill that responsibility. So, I felt a little self-pressure.” [26 years, PTSD–/HIV–]	“Sometimes I’d be doing something on the street and my phone would go off and tell me to complete a survey. It was a little inconvenient.” [20 years, PTSD–/HIV–]	“Usually there are no problems, but if in the course of the day, you ask me to do too many surveys, I will get lazy and won’t want to do any more...For example, after I finish [a survey], sometimes just an hour or two later I’d receive another prompt. Basically, it felt like I just completed a prompt and since that time, nothing has changed. So, I won’t do the other survey.” [24 years, PTSD+/HIV+]
Usability	“I had difficulty uploading the audio file on the app. But then I called [a research staff] and she showed me how to...then it was fine.” [28 years, PTSD–/HIV–] “I am okay with doing the surveys [for however long]...They are easy. Whenever I receive a prompt, I always do the survey right away. Because of [the nature of] my work, I’m always free.” [25 years, PTSD–/HIV–]	“I liked the file recording more than the surveys because it’s like you are speaking to someone, speaking directly to someone else. For example, when I want to share something with a friend, I’ll also [send audio files]. Every time I’m down, I’m more likely to audio record than message. The audio file is like that, like chatting with someone.” [20 years, PTSD+/HIV–]	“Normally, the surveys are pretty easy to complete, but the problem is that I am busy. If I am not busy, then I can complete them easily. But on days where I work, often I won’t get up until 10AM. I know I should complete the morning survey, but unfortunately, that might also be when I have a customer and I might not finish until 2-3PM. Then suddenly I’d receive the second survey and I won’t know what order I should reply in.” [29 years, PTSD+/HIV–]
Recommendation	“[It would be more reasonable] if there were prompts every 3-4 hours, for example, one survey in the morning, one survey at night, and possibly two surveys in between. [As is, it] is too much.” [27 years, PTSD–/HIV–]	“I just want all the emotion questions to be combined into one, a ‘how do you assess your emotional state right now?’ question. [This is] because sometimes I don’t experience any emotion strongly...and it’s hard to answer.” [20 years, PTSD+/HIV]	“I think you can shorten the questions because many of them are very similar. They can just be combined.” [24 years, PTSD+/HIV+]

^a^PTSD: posttraumatic stress disorder.

### Qualitative Results

#### Relevance

Overall, participants considered questions about their social interactions (eg, perception of the person), mood, levels of stress, and health, including their experience of PTSD symptoms and medication use, most relevant to their daily experiences. Participants favored these items as they thought these items provided a dedicated space for daily reflections and made several participants feel as though someone cared for them. For these reasons, the audio recording was often singled out as the most meaningful survey component as it allowed participants to elaborate, in their own words, what had transpired that day:

[After the recording], I felt a lot more comfortable, as though there was nothing more I wanted to share. Whenever I can share everything I want, I will feel lighter. [My mind] still feels heavy, of course, but my mental state will feel better than if I were to just dwell on it and keep it inside.

20 years, PTSD+/HIV–

In some cases, the audio recording was seen as even therapeutic as it allowed participants to share distressing thoughts or feelings that they otherwise would have concealed. One of the participants contrasted the freedom he exercised in sharing details of his life in the app with the caution he felt when disclosing to friends:

[I] feel reluctant to be open, to share with others. You see a lot of surveys because I shared everything I had, because in my heart, I think that, first, you’re a stranger, and second, you won’t affect my life. So, I can say all those things comfortably, and even more. But when I must talk about these issues to my friends or co-workers, it becomes very difficult, very different. You can be very close to a colleague and think of them as a close friend, but when they’re not able to keep your secret, then a second person [might know]...In their mind, they might think they’re just sharing with a close friend of theirs, but that close friend might share with another close friend, then another close friend, then a rumor circulates. So, I have this cautiousness towards those whom I interact with.

25 years, PTSD+/HIV+

Questions that were considered less relevant to participants’ everyday experiences were generally centered on alcohol or drug use. Although a few participants reported drug use in the past 6 months, most felt that their use was not frequent enough for either drug use or drug craving to be repeatedly sampled:

I’m not someone addicted to those drugs so most of the time I won’t be affected by them. It’s only sometimes when I feel like using them, and when I do, it’s only for fun. So, I think those questions are irrelevant to my life.

24 years, PTSD+/HIV+

Others thought that despite their infrequent use of substances, the questions were still relevant as they were still exposed to drug-related activities in their everyday lives. Several participants felt that they should be able to document those events as those exposures can make them feel uncomfortable or increase their desire to use.

As many questions focused on PTSD and negative affect, some participants felt that the prompts were more appropriate for someone in greater psychological distress than them. For this reason, some suggested the addition of more positive questions (eg, highlighting coping strategies or health-promoting behaviors). Furthermore, as multiple questions measured the same construct (eg, symptoms of distress), items were sometimes seen as repetitive and requiring a more careful read by participants. Some participants believed that the emotion measures, for instance, required them to parse their affect too finely. Instead of having items such as *hopeless* or *happy*, several participants favored using a Likert scale ranging from *negative* to *positive*:

Questions like “happy” or “proud” are probably okay because people can separate between the two, because those emotions are different. But the questions that are more negative, [you should] reduce those because if someone is already thinking negatively and they keep reading those questions, it’ll also drag their mood down more.

28 years, PTSD+/HIV+

#### Benefits of Using the App

Nearly half of those who used the app said that they believed it increased their self-understanding. In particular, many believed that using the app enabled them to better understand their emotions and the individuals or situations that elicited those feelings. In doing so, some participants believed that the app made them more truthful about their emotions and what specifically they had accomplished that day:

When I do the surveys, I realize that sometimes I have symptoms that I didn’t notice before; like, an hour ago I might have felt calm, I might have felt anxious, or I might have felt something else...Before I wouldn’t notice these things. Since I started doing the surveys...I [started thinking] to myself that, yes, I do have these symptoms, symptoms I didn’t notice before.

23 years, PTSD–/HIV+

With this increased self-understanding, a few participants believed that an additional benefit of using the app was that it enabled them to better monitor and regulate their emotions over time, that is, to identify what they could control and what they might need to change. For some, this form of self-understanding formed the basis of their desire for greater self-care:

I know how to control my emotions more because if every 2-3 hours I have to answer the survey, then automatically, I have to think about whether I was happy, sad, stressed, or whatever else in the past hour. When I answer those questions (for myself), then I have to reassess how the situations were affecting my mood. So, I can control those factors better.

25 years, PTSD+/HIV+

Those who reported no new self-understanding or benefit often found the questions too reflective of their everyday life and therefore could not offer new insights:

It’s because normally I am someone who understands themselves well already, so your app has the effect of helping me determine what I’m feeling at an exact moment. After that, I can adjust it myself so that [my mood] slowly goes back to being normal. But to say that I understand more about myself, I don’t think I did.

25 years, PTSD+/HIV+

#### Worries and Concerns

Participants reported a few worries or concerns associated with using the app as they perceived the survey as similar to other questionnaires they had completed before and because of the detailed briefing they received before starting the study:

Actually, [the surveys are] like a test, or an assessment. They don’t cause any problems or concerns. That’s because it’s...it’s like you’re just monitoring your health, doesn’t really concern much else.

25 years, PTSD+/HIV+

I mean, I already found out all the information before I agreed. For instance, if this were PrEP, even if you gave me a lot of money, I still wouldn’t. The truth is I only do the things that are good for me. I’m not going to be a test mouse. Psychological questions are fine because it doesn’t really affect me in any way, but PrEP, that affects my health.

26 years, PTSD–/HIV–

Others expressed privacy concerns related to the accessibility of their surveys. In particular, some participants worried that others might see their screen while they were answering sensitive questions about their sexual behavior or medication use, although only one of the participants experienced a situation in which someone had seen his responses:

On one of the days [I participated], my nephew was being naughty and took my phone from me. It was also right when I had been filling out a survey. He read some of the questions and asked what I was doing, why there were questions related to marijuana, drug use, all that. I had to sit him down and explain to him that I was participating in this study at Hanoi Medical University, that it wasn’t anything serious...In this situation, I knew how to resolve it, but for others, it might create a misunderstanding.

24 years, PTSD+/HIV+

#### Usability

Participants found completing the initial EMA survey with a research staff helpful. Most participants did not experience any difficulties in navigating the EMA form. Participants struggled the most when uploading an audio file. Depending on the model of their phone, some were not able to record and upload directly onto KoBo but had to use a third-party platform to save the files. Others were also initially confused about the kinds of files they could upload and submitted photos instead.

When asked whether they would use the app outside of the study, most participants said that they would but that their use would depend on how busy they were. Some emphasized that their use of the app was largely motivated by their current interest in mental well-being:

It depends on the time-point. At this time-point, I’m very concerned about my mental health, and whenever I finish a survey, I always re-ask myself the questions. While I am still confronting these [mental health] problems, I will answer the prompts because they help me identify what works for me. In the future, when I no longer have these problems, I will think that [completing the questions] isn’t important, that it is fine to respond, fine not to respond. I won’t care anymore.

25 years, PTSD+/HIV+

When asked what features would encourage their long-term use of the app, a few said that they would feel more encouraged to use the app over a longer period if they could see daily or weekly trends in their responses or were given personalized recommendations to improve their health or general well-being (eg, sleep tips and coping strategies). Most participants emphasized that their continued use depended less on new features than on changes to the existing study design, including the number and scheduling of the prompts:

When I am available, I will do it. When I am not, then I won’t do it because it’ll be too much. But if I’m free, then what else am I doing but playing [Candycrush]?

23 years, PTSD–/HIV–

Others thought that their willingness to answer the prompts was variable throughout the day, depending on their mood:

When I am tired, I’ll be truthful and say that I don’t want to do [anything]...There are times when I don’t want to do anything, let alone a survey taking 5-10 minutes, ticking off questions after questions. And, as I’ve said, it eventually gets boring when you have the same [set of questions] every day. I also think that [reception to] the survey will depend quite a bit on the psychological state, the mood, of the person taking it.

25 years, PTSD+/HIV+

#### Recommendations

When asked to provide recommendations to increase future compliance and better tailor content to their everyday experiences, 5 main themes emerged. First, there was an emphasis on supplementing the initial training with detailed reminders on how to use the app, as the participants reported initial confusion when completing the first few surveys on their own and having to contact the study staff for assistance. Second, the wording of the daily prompts and study questions should be made as personable as possible. Although the current phrasing was deemed easily comprehensible, it was also seen as too clinical. Where possible, the participants thought the text should be more MSM friendly, prompting people to share (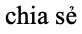) their everyday experiences (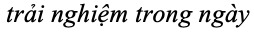) rather than strictly completing a survey. Having interactive features was also found to be critical in increasing engagement.

Third, although open-ended questions took more time to answer, they were considered more experientially relevant than the Likert scale responses. Integrating optional open-ended questions throughout the day was favored over having only the audio recording before sleep. The option to type rather than narrate the responses was considered very important, as some participants thought that the audio recording was inconvenient when others were nearby. Although generally preferring open-ended questions, participants also felt that all survey items, including the audio recording, should be optional. Reflecting on his experiences, one of the participants shared his belief that many Vietnamese people needed to be eased into doing the audio recording as it did not come naturally:

It’s because people feel embarrassed. Why? The audio recording is like a diary, except you write in a diary; here you have to record, retell. It’s [related to] personality type, like, you might feel embarrassed because you’re more introverted. It makes you feel autistic (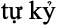). Personally, I feel too embarrassed to say aloud my inner thoughts. Writing is okay, but I fear sitting alone and talking aloud like that because when I do I feel as though I’m being autistic (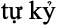). Singing is okay, but talking aloud like that is similar to splitting yourself in half [to sit there, look at yourself, and judge yourself].

26 years, PTSD+/HIV+

Fourth, most participants found the number of assessments acceptable but took issue with their scheduling. Although participants were told that they did not have to answer the surveys when busy, many reported feeling obliged to do so and actively monitoring their phones for prompts. Participants recommended sampling only once or twice during prime hours and potentially lengthening the time frame they had to respond. This was seen as particularly useful when participants’ schedules deviated from those they provided at enrollment, which often happened because of the types of work young MSM were engaged in. Finally, participants recommended reducing the number of questions to prevent future participants from answering the prompts haphazardly (cho nó qua). This could be done by combining similar questions, occasionally changing the order of questions to make the surveys more interesting, and introducing new questions. Having access to aggregate data, particularly those that compared participants with the group average, was seen as potentially motivating, alongside receiving custom health information:

[The app] keeps asking these questions, everyday these same questions. I think it needs to be a little different, so there’s enthusiasm to answer.

28 years, PTSD–/HIV–

## Discussion

### Principal Findings

This study found that mEMA is a feasible and acceptable way of monitoring dynamic interactions between mental distress and HIV risk among young MSM in Vietnam. Overall, participants found the questions pertinent to their everyday experiences of psychosocial stress and were willing to use the app in the future. Participants identified items related to PTSD symptoms, emotions, and social interactions as most relevant to their everyday experiences. Most preferred having open-ended questions to elaborate on their stressful encounters, as they found doing so therapeutic or personally informative. Some expressed concerns about data privacy. Although the number and length of EMA surveys were considered potential hindrances to long-term participation, the impersonality of the app and the scheduling of the prompts rather than their frequency were identified as primary barriers to participation. Participants recommended that the language be made more MSM friendly and that future iterations of the app include personalized end-of-the-week reports to encourage long-term use.

Given that our sample was highly exposed to trauma, identification of barriers to feasibility and acceptability was important. Our compliance rate was noticeably lower than those reported previously in similar studies with MSM [71-73]. Although our assessment period was relatively shorter (1 week vs 4 weeks), we sampled participants more frequently and included more questions because of our interest in acquiring a thorough understanding of how PTSD interacts with HIV risk. This may have reduced the compliance rate. In addition, young MSM may introduce unique challenges. A recent meta-analysis of EMA studies found that age was the most significant predictor of compliance rate [74]. In particular, although the response generally declined over time, this decline was steeper among younger participants compared with that in older participants. Smiley [75] reported a response rate (57.3%) similar to ours among young gay and bisexual men (aged 21-25 years, which they attributed to the participants’ work schedules. In the context of Vietnam, these issues may be further exacerbated, as several participants had highly variable work schedules. For instance, one of the participants did not answer any of the EMA prompts as shortly after enrolling in the study, he was assigned to a mining post in rural Northern Vietnam for the study duration and had limited access to his phone. Cases such as these lowered our overall response rate but may accurately reflect the challenges typical in implementing EMA protocols in LMIC contexts.

Incentivization structures can motivate compliance. Doherty et al [76] highlighted the use of extrinsic and intrinsic incentives to motivate participants’ EMA engagement. Extrinsic motivation includes monetary remuneration per survey, compliance monitoring and feedback, and emphasis on participants’ contributions to science during enrollment. In particular, providing feedback to participants during an EMA study, whether through reminder messages or weekly reports, has been found to increase their perception that someone cared and thereby motivate compliance; however, this may also contribute to reactivity [77]. In addition, although financial incentives are useful, they can also contribute to selection bias [76]. This was a concern that our participants also voiced, as some thought that the length of the survey, alongside financial incentivization, might encourage participants to answer haphazardly (cho nó qua). Coupling extrinsic incentives with features that cultivate intrinsic motivation may reduce these unwanted effects. These features enhance or support participants’ inner desire to engage with the app, independent of momentary awards. For instance, Hsieh et al [78] found that participants who had access to visualizations of their data had a 23% higher compliance rate than those who did not. Similarly, the attitudes of research staff during enrollment and participants’ ability to access their own response rate have been shown to motivate participation [79]. In our study, interest in mental health was a particularly strong motivator. Participants also perceived having an MSM-friendly language as highly important. A recent meta-analysis of mHealth interventions for young sexual and gender minorities suggests that these linguistic issues are often not well attended to; across studies, the most common concerns centered on language, specifically on prompts that were seen as too text-heavy, patronizing, or superficially targeted toward LGBTIQ+ (lesbian, gay, bisexual, transgender, intersex, and queer) people [80]. Among our study participants, an intrinsic motivation evidenced throughout multiple interviews was the desire to better understand and regulate their mental and emotional states.

Our concern that mental health stigma may lead participants to underreport experiences of mental health and psychosocial distress was largely unfounded [81-83]. For some participants, mental health stigma may have even enhanced the perceived attractiveness of the app, as it provided them an outlet to share details they otherwise could not. These findings underscore the value of testing assumptions regarding acceptability *in the wild*. Most of our participants readily looked to the app for mental health and psychosocial support information, which is encouraging for digital mental health interventions. However, this inadvertent reliance on the app for mental health and psychosocial support contributed toward a second set of concerns about reactivity or when the frequency, intensity, or quality of a target measure change as a result of monitoring [84]. Specifically, several participants reported that they intentionally used the app to track and regulate their emotional states; some reported becoming more aware of everyday triggers. Whether this altered the experience of mental distress or the relationship between mental distress and HIV risk behaviors is unclear. Variable effects have been documented in the literature. Although some studies report an improvement in mood or change in risk-taking behavior among participants using EMA [85,86], others have found no significant reactivity effect [84,87,88]. Given the scope of this study, we were not able to determine whether the frequency, intensity, and correlations between measures varied significantly over time.

Responses to the survey further highlighted some of the cultural specificities that might contribute to differences in the perceived acceptability of an app in different populations. Notably, our sample strongly preferred open-ended questions about their life but disliked the option to self-record as they found the behavior—when done alone—to be indicative of 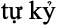 (roughly means “autism”), which is highly stigmatizing within the country. As mHealth and telehealth approaches are becoming more widely adopted worldwide [89], the question of how to culturally adapt and enhance the acceptability of these applications has become imperative. However, caution must be exercised in interpreting differences as necessarily cultural in nature. The feedback we received on the assessment of emotional states is illustrative: studies of emotion worldwide have demonstrated cross-cultural differences in emotional granularity or the degree to which individuals differentiate emotional states and identify them with precision and specificity [90]. Anthropological investigations of emotional experience worldwide have further demonstrated how conceptualizations and management of emotions differ worldwide (eg, are localized to the heart or seen as inseparable from thought) [91,92]. These differences point to the need not only to investigate the cultural appropriateness of EMA measurement approaches during the app design and evaluation process but also to evaluate alongside this the degree to which experiences of the app reflect differences based on mental health status. Specifically, emotional granularity has been shown to be reduced in a number of mental health conditions, including PTSD [93]. It is possible that those with higher PTSD symptom severity may experience greater difficulty in recognizing, differentiating, and labeling the different emotions they experience. Building in the means to tease apart the potential causes of difference is critical to tailor EMA protocols appropriately to local contexts and populations.

### Study Strengths and Limitations

This study is innovative in several ways. To our knowledge, this is the first study to examine the feasibility and acceptability of using mobile technology to model the dynamic relationships between mental distress and HIV risk in a low-resource setting, and it is the second EMA implemented in an LMIC [54]. The study demonstrates the potential of leveraging such technologies not only to complement existing prevention and intervention strategies but also to advance EMIs that cater to the dynamically changing risk of individuals across time and context. Furthermore, compared with similar feasibility and acceptable studies, the sample size for this study was larger than those that have been conducted among high-risk populations such as MSM, people living with HIV, and people with psychiatric disorders [71,73,94], allowing for a more granular analysis of how adherence to, tolerance of, and experience with EMA protocols differ in this population. Recruitment of individuals with both PTSD and HIV proved particularly challenging not only because many refused to participate but also because many either had physically relocated or been unreachable at follow-up. Of the 4 individuals who withdrew from the study, 3 (75%) were also from this group. Although it is not possible to discern the reasons that motivated this decision, future research should investigate how psychiatric comorbidities of HIV may affect the receptiveness of MSM to varying demands of mEMA research and explicitly assess the reasons for dropout across those different study designs.

The primary limitations of this study include the generalizability of the findings and potential reactivity. First, although the sample size was larger than comparable studies, participants were recruited from community-based organizations and clinics and, thus, present a subpopulation in Hanoi that accesses sexual health services and are engaged in care. Participants were additionally drawn from a previous study among those who had agreed to be recontacted; therefore, these individuals may be more inclined to volunteer in research. Compared with the national average, our sample was also more highly educated and had a higher income [95,96]. Concomitantly, all our participants owned a smartphone and, therefore, may represent a subpopulation that is more comfortable using mobile apps than their counterparts. This is unlikely to be representative of older Vietnamese MSM in urban settings and those in the country more generally among whom HIV risk and mental health burden may be both more stigmatized and pronounced [83,97,98]. Thus, the generalizability of these findings to other MSM should be taken with caution. Second, although participants noted that they had become more aware of their emotions and were using self-reports to monitor and regulate aversive triggers, it is inconclusive whether using the app actually changed the frequency with which they engaged in risk-taking behaviors or experienced particular PTSD symptoms or emotions. Previous EMA research has found minimal reactivity to EMA [84,99]; however, given the stigma associated with both HIV and mental health issues within Vietnam, sharing otherwise concealed details of their lives to the app may have heightened therapeutic effects.

### Conclusions

Our study illustrates the feasibility and acceptability of mEMA studies among high-risk Vietnamese MSM. Participants were receptive to and invested in future studies and interventions. The findings of this study can inform the design of future EMA studies to optimize relevance, usability, and acceptability. Achievement of the Joint United Nations Program on HIV/AIDS [100] 95-95-95 targets by 2025 requires the integration of such novel technologies with existing prevention and intervention efforts. Given their ubiquity, mEMA studies hold tremendous promise for furthering our understanding of the proximal mechanisms potentiating HIV risk, including the contextual and psychological conditions under which they occur, which are conditions difficult to recall later on. Such information is essential for the design and delivery of EMIs that can efficiently and effectively provide resources to when, where, and whom they are most needed.

## Abbreviations

ART: antiretroviral therapy
CMD: common mental disorder
EMA: ecological momentary assessment
EMI: ecological momentary intervention
LGBTIQ+: lesbian, gay, bisexual, transgender, intersex, and queer
LMIC: low- and middle-income country
mEMA: mobile ecological momentary assessment
mHealth: mobile health
MSM: men who have sex with men
PrEP: pre-exposure prophylaxis
PTSD: posttraumatic stress disorder
